# Perception of threat and intent to harm from vocal and facial cues

**DOI:** 10.1177/17470218231169952

**Published:** 2023-05-10

**Authors:** James Tompkinson, Mila Mileva, Dominic Watt, A Mike Burton

**Affiliations:** 1 Aston Institute for Forensic Linguistics, College of Business and Social Sciences, Aston University, Birmingham, UK; 2 School of Psychology, University of Plymouth, Plymouth, UK; 3 Department of Language and Linguistic Science, University of York, York, UK; 4 Department of Psychology, University of York, York, UK

**Keywords:** Voice perception, threat, intent to harm, audiovisual integration

## Abstract

What constitutes a “threatening tone of voice”? There is currently little research exploring how listeners infer threat, or the intention to cause harm, from speakers’ voices. Here, we investigated the influence of key linguistic variables on these evaluations (Study 1). Results showed a trend for voices perceived to be lower in pitch, particularly those of male speakers, to be evaluated as sounding more threatening and conveying greater intent to harm. We next investigated the evaluation of multimodal stimuli comprising voices and faces varying in perceived dominance (Study 2). Visual information about the speaker’s face had a significant effect on threat and intent ratings. In both experiments, we observed a relatively low level of agreement among individual listeners’ evaluations, emphasising idiosyncrasy in the ways in which threat and intent-to-harm are perceived. This research provides a basis for the perceptual experience of a “threatening tone of voice,” along with an exploration of vocal and facial cue integration in social evaluation.

## Introduction

Human voices and faces are some of the richest sources of social information in our everyday lives. We often use them to infer others’ age, sex, identity, or emotional state as well as a range of socially relevant traits such as approachability, confidence, and intelligence (Belin et al., 2011; Bruce & Young, 1986, 2013; McAleer et al., 2014; Oosterhof & Todorov, 2008). These latter judgements are often informed by transient cues or states (e.g., this person is angry right now), which are then overgeneralised as signals of stable traits (e.g., this is an aggressive person, Zebrowitz & Montepare, 2008). Social judgements based on both voice and face cues have been shown to follow the same two-dimensional structure with valence (trustworthiness) and dominance as the two fundamental dimensions (McAleer et al., 2014; Oosterhof & Todorov, 2008, but see also Sutherland et al., 2014). The evaluation of threat is considered to be at the core of first impression judgements with valence (trustworthiness) being a signal of someone’s intent to cause harm and dominance being a signal of someone’s ability to implement such harm (Todorov, 2008; Todorov et al., 2008; Zebrowitz et al., 2010). Despite this, current first impression models have generally not included threat as a trait of interest or importance (McAleer et al., 2014; Oosterhof & Todorov, 2008). Oosterhof and Todorov (2008) show a strong negative correlation between ratings of threat and trustworthiness and a strong positive correlation between ratings of threat and dominance. It is therefore likely that the evaluation of threat is associated with both trustworthiness and dominance. However, the theorised associations between trustworthiness and intent to harm on one hand and dominance and ability to harm on the other, have not been systematically examined.

Threat detection is also of high evolutionary importance, with evidence showing that reliable impressions of threat based on neutral face images (i.e., those not showing an obvious emotional expression) can be formed faster than other social traits such as intelligence (Bar et al., 2006) and can even be detected non-consciously in expressive faces (Pessoa et al., 2005; Williams et al., 2004). Moreover, while first impression judgements have been shown to influence our political, economic, and court sentencing decisions (Ballew & Todorov, 2007; Chen et al., 2016; Mileva et al., 2020; Tigue et al., 2012; Wells et al., 2009; Wilson & Rule, 2016), the perception of threat could have even more serious legal consequences. For example, it is not illegal to be perceived as dominant but it might be illegal to be perceived as threatening. It is, therefore, surprising that we know relatively little about how threat can be conveyed in the human voice and face. This could lead to potential problems when those tasked with assessing threats are forced to rely on their own assumptions rather than findings from empirical research (Gales, 2017).

Linguistic research on threatening language has predominantly focussed on threats as speech acts, the classification of different threat types (Al-Shorafat, 1988; Fraser, 1998; Gingiss, 1986; Storey, 1995; Yamanaka, 1995), and the analysis of specific linguistic features in written threats (Carter, 2010; Gales, 2011, 2012, 2015; 2017). For example, research on written threats identifies the presence of the modal verb “will” in its noncontracted form, for example, “I will hurt you” or “I’m warning you, it will end badly if you don’t comply,” as a potential facilitator to the perception of threat (Gales, 2010). Not only do such prediction modals emphasise the certainty and commitment on the part of the threatener (Nini, 2017), but they have been noted as a characteristic feature of higher-level, more credible, threats (Gales, 2017; Napier & Mardigian, 2003). Threats are a rare form of speech act as the verb “threaten” is hardly ever used performatively in English. For example, it would be very unlikely for a speaker to make a threat using the construction, “I’m threatening you one last time.” However, it is perfectly possible for threats to take the same constructions as other types of speech acts such as warnings, with an utterance of the type “I’m warning you one last time” interpretable as a threat given the correct context. Threats are also frequently heard and evaluated by listeners who are not the intended recipients of the threatened action. This can occur both at the time of the delivery of the threat, such as in the context of an emergency call handler receiving a bomb threat in a 999 call, or after the event, such as in the context of a jury panel evaluating a potential threat during a criminal trial.

Few studies have investigated how aspects of speakers’ voices could affect listeners’ perceptions of spoken threats. Watt et al. (2013) state that a speaker’s “tone of voice” can be used in the legal system to refer to aspects of speech that listeners may use to infer threat. Milburn and Watman (1981) argue that the meaning of threat utterances and the inferences that listeners make from them can change depending on the speaker’s tone of voice. Investigating this further, Watt et al. (2013) found that listeners inferred greater levels of threat from productions of the indirect threat, “I know where you live” when it had been designed by the speaker to sound threatening, compared with productions of the same sentence that had been designed by the speaker to convey no threat or intent to harm. This challenges the idea that only the words used in a spoken threat can influence either its meaning or interpretation, particularly when the utterance in question is indirect, vague, or could be interpreted as another type of speech act. Nevertheless, the assumption that both a speaker and a hearer will “know a threat when they hear one” has been considered “the majority view” despite its rather obvious insufficiency for courtroom, legal, or investigative purposes (Gingiss, 1986).

Research on first impression judgements can be used to reveal more about the phonetic parameters of threats. This is primarily due to the idea the evaluation of threat forms the basis of the underlying first impression dimensions, trustworthiness, and dominance, as well as the traits that come together to shape these dimensions (McAleer et al., 2014; Oosterhof & Todorov, 2008). A key finding in this literature is the relationship between lowered Fundamental Frequency (hereafter F0) and perceptions of increased social and/or physical dominance (Mileva et al., 2018; Ohala, 1984; Puts et al., 2006, 2007; Tusing & Dillard, 2000). F0 is a measurement of the rate at which the vocal folds vibrate, and is measured in Hertz (Hz) as the number of vocal fold vibrations per second. F0 is an acoustic correlate of vocal pitch, with lower-pitched voices having lower F0 and higher-pitched voices having higher F0 measurements. However, one-to-one correspondence between these two features should not be assumed as other aspects of the voice, such as voice quality, can influence the perception of pitch. Intonation, that is, variation in the F0 contour, has also been identified as a phonetic marker of speakers’ attitudes and emotions including arousal, anger, joy, or doubt, along with aspects of speaker intention (Vaissière, 2005). Moreover, McAleer et al. (2014) identify a number of acoustic properties such as F0, intonation, harmonic-to-noise ratio and formant dispersion as significant predictors of the two underlying social evaluation dimensions.

Regional accent is another indisputably important element of how listeners evaluate and form attitudes towards speakers (see Watson & Clark, 2015 for a review), with studies generally illustrating that identities with standard accents are perceived as more intelligent, of higher social class and more attractive, but also as potentially more aggressive and less kindhearted than identities with non-standard accents (Coupland & Bishop, 2007; Giles, 1970; Giles et al., 1981). Regional accents can be particularly important in evaluations that take place in legal settings (Kalin, 1982), where judgements about speakers can potentially have severe consequences. Experimental work testing the effect of regional accents has highlighted that speakers with non-standard accents are more likely to be perceived negatively in matched-guise mock juror experiments (Dixon & Mahoney, 2004; Dixon et al., 2002). Non-standard accents have also been shown to convey greater levels of threat for indirect threat utterances compared with standard varieties (Tompkinson, 2015).

The need for more linguistic research on the perception of spoken threats gains further importance in the legal context where threats can be seen as criminal acts. For example, a UK parliamentary report highlights threat perception as a potential source of disconnect between how linguistic experts view speech analysis compared with members of the public (Bunn & Foxen, 2015). It states that jurors expect procedures such as personality analysis, determining truth and falsity, and assessing threat in speech intonation to be possible, despite the fact that experts assert that they are not currently able to do these things. The last point in this list highlights unrealistic juror expectations and beliefs that various aspects of the human voice can be used to determine threat and intent-to-harm, with the latter of these being particularly important in the criminal context (Watt et al., 2013).

One of the key characteristics of social judgements, and therefore of threat evaluations, is that perceivers tend to agree with each others’ impressions, making them highly reliable. This has been consistently demonstrated with judgements purely based on the acoustic information from the voice (McAleer et al., 2014; Rezlescu et al., 2015; Zuckerman & Driver, 1989) as well as on the visual information from the face (Kramer et al., 2018), although the latter has been shown to achieve a somewhat higher level of inter-rater agreement (Lavan et al., 2021). This agreement has been consistently demonstrated across different age, race, and culture groups of raters and for both traits with social (e.g., warmth, attractiveness, and intelligence) and more evolutionary (e.g., trustworthiness and threat) importance (Albright et al., 1997; Cogsdill & Banaji, 2015; Zebrowitz et al., 2012). This reliability should not be conflated with accuracy, however, rather a measure of how consensus exists among perceivers. Despite all of this, a constantly growing number of studies have raised concerns about the way this agreement has been traditionally measured, namely using Cronbach’s alpha. Many have now pointed out that this measure could potentially lead to overestimating actual agreement (Cortina, 1993; Dunn et al., 2014; Green & Yang, 2009; Kramer et al., 2018), which could have serious implications, especially when it comes to dimensions with direct relevance to legal and judicial contexts such as perceived threat.

### Research aims

Here, we present two studies which address the question of how listeners infer threat and intent to harm from voices (Study 1), and from the combination of vocal and facial cues (Study 2). Across the two studies, we examined the influence of a range of parameters, including median F0, intonation (F0 range), speaker accent, emphasis pattern, type of utterance (warning vs. statement), perceived pitch, perceived speech speed, and speaker sex. As the perception and interpretation of threats are critically important in the applied context, we also explored the agreement among listeners on the levels of threat and intent conveyed by each speaker. We opted to assess perceptions of both threat and intent to harm following the rationale set out by Watt et al. (2013) that adequate demonstration of intention is necessary for a threat to qualify as a criminal offence under UK law. This intention can be either actual intent on the part of the threatener, or perceived intent on the part of the listener. In Study 1, participants were presented with simulated bomb threat calls and were asked to provide threat and intent to harm ratings for each speaker and in Study 2, we explored the integration of vocal and facial cues by allowing participants to see a face alongside hearing the voice. We were particularly interested in the effect of facial dominance as a threat cue and paired each voice recording with two different images of the same speaker—one that had been pre-rated as high and another that had been pre-rated as low on the dominance scale.

Based on the close links between F0 and the perception of dominance and threat judgements, we expect that utterances with lower F0 and those with lower perceived pitch will be perceived as conveying higher levels of threat and intent to harm. Given previous research on written threats, it is also possible for utterances stressing the modal verb “will” to be perceived as more threatening and intentful than utterances stressing other words in the sentence (Gales, 2010; Nini, 2017; Tompkinson, 2018). The current accent literature presents some inconsistent findings, but given that non-standard accents have been shown to be less favourably perceived in mock jury experiments, we anticipate higher ratings of threat and intent to harm for non-standard accents in our study compared with the standard accents. In addition, rather than adopting a matched-guise design, as has been used in other research on accent evaluation in legally relevant research (Dixon et al., 2002; Dixon & Mahoney, 2004), we use genuine speakers of each of the tested accents in an attempt to more accurately simulate real-world voice evaluation situations. Finally, for Study 2, we expect that voice recordings paired with a dominant-looking image of the speaker will be perceived as more threatening than those paired with a non-dominant face image and that this effect will be present for ratings of threat, but not intent to harm since the trustworthiness, not the dominance, dimension is thought to reflect evaluations of intent.

## Study 1: perceptions of threat and intent to harm in voices

In our first study, we assessed the relative effects of different aspects of voice on listeners’ perceptions of threat and intent to harm. The goal of the analysis was to gain a greater understanding of listeners’ decisions about what makes a speaker sound threatening and intentful, and to assess the roles that different aspects of voice appear to play in shaping these decisions. We opted to use an experimental design which aimed to mirror the type of evaluations that a juror might be asked to make about a potential threat, rather than a design where participants were instructed that they were under any kind of threat. The evaluations made by listeners were therefore undertaken in the role of a third-party evaluator. We considered this to be the most appropriate design given that it is the task of jurors to evaluate this kind of evidence in court, and the approach did not place undue burdens on our participants. Furthermore, juror behaviour cannot be assessed directly, leaving experimental approaches as one practical way to evaluate human behaviour in this context.

### Method

#### Participants

A total of 85 participants (9 male, median age = 19, age range = 18–55) took part in the experiment. All participants were students at the University of York and were native British English speakers. They were all tested in either the Department of Psychology or the Department of Language and Linguistic Science at the University of York and received payment or course credits for their participation. Sample size was based on previous work, showing that a sample of 20 participants is sufficient to produce a stable mean rating of both faces and voices as well as a significant level of rater agreement (Lavan et al., 2021). Each listener was asked to evaluate a sub-sample of voice recordings, with an average of 20 listeners rating each individual utterance. Informed consent was provided prior to participation and experimental procedures were approved by the ethics committees of the Department of Psychology and the Department of Language and Linguistic Science at the University of York.

#### Materials

The experimental stimuli comprised 48 voice recordings produced by 12 student volunteers (6 male, age range = 18–30). Speakers provided informed consent to be recorded producing the utterances “There’s a bomb at York Station. It will go off this afternoon” and “I’m warning you about a bomb at York Station, which will go off this afternoon.” The stimuli were constructed using commonly-found features in real-world threats (Gales, 2010; Napier & Mardigian, 2003; Nini, 2017). These included the use of utterances which had potential alternative interpretations as either a warning or a statement of fact, talk of a violent act (in this case the detonation of a bomb), and utterances in which the violent act was directed towards a third-party rather than the direct recipient of the utterance. Speakers were instructed to produce each utterance twice, once with emphasis on the word “will” and once with emphasis on the word “this.”

Recordings were conducted in a quiet environment using a Zoom H4N handheld recorder with a built-in microphone. This was placed on a table approximately 30 cm from each speaker. Four speakers were self-identified speakers of Standard Southern British English (SSBE), four were self-identified speakers of Northern Irish English, and four were self-identified L2 speakers of English who had languages of the Middle East as a native language (three Arabic speakers, one Persian speaker). We acknowledge that there are differences within the speakers of our L2 accent group, with Arabic and Persian clearly being different and distinct languages, but we grouped them together in this experiment to distinguish speakers with English as their first language from those with English as a second language. The rationale for the grouping is the same as that set out in Tompkinson and Watt (2018), who explain that the differences in the English of these L2 speakers was not distinct enough to create clear differences in speakers’ evaluations of the accents. Within each accent group, there was an equal number of male and female speakers. The recordings were 16-bit, single channel digital audio recordings with a sampling rate of 44.1 kHz. Once recorded, the acoustic stimuli were transferred onto a computer hard-drive at the original sampling rate and subsequently band-pass filtered between 300 and 3,400 Hz to simulate the landline telephone channel (Künzel, 2001; Nolan et al., 2013). Band-pass filtering was conducted using Praat’s (Boersma & Weenink, 2016) in-built filtering function. A 0.5-s period of silence was added to the end of each utterance, and this was followed by a 1-s long 175 Hz tone which was designed so as to resemble the hang-up tone that signals the termination of a call. This was, again, conducted using Praat (Boersma & Weenink, 2016).

#### Design and procedure

**Figure 1.**
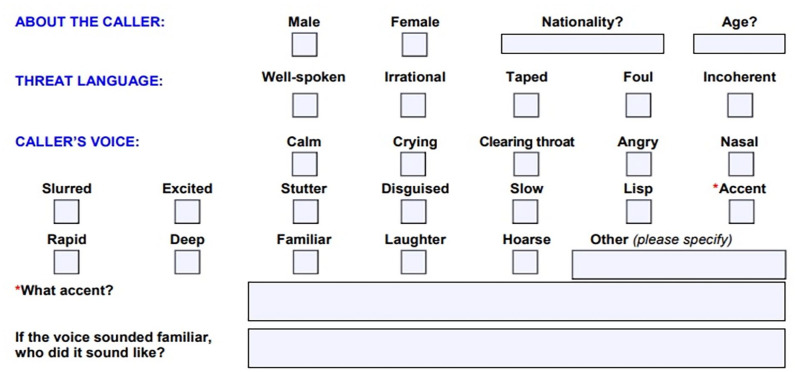
Extract from UK National Counter Terrorism Security Office bomb threat checklist relating to aspects of speakers’ voices.

The experiment was created and hosted on the online platform Qualtrics (Provo, UT); however, participants completed the task in a computer lab, in person. All participants were presented with a randomly generated subset of vocal stimuli in order to avoid any learning and familiarity effects as the stimuli were produced by 12 speakers only. The mean number of listeners who provided threat and intent ratings for each utterance within the experiment was 20. We investigated the relative influence of a range of parameters on listeners’ threat and intent evaluations. These included *median F0* as an average measure of how high-pitched a speaker’s voice was; *F0 range*, as a measure of how much intonational variation was present in each utterance; *speaker accent* (SSBE, Northern Irish, Middle Eastern); *emphasis pattern* (emphasis on “will”/emphasis on “this”) and *utterance* (“I’m warning you about a bomb . . . ”/“There’s a bomb . . . ”). We also explored the effect of *speaker sex*. For male speakers, the pitch range in Praat (Boersma & Weenink, 2016) was set between 75 and 300 Hz, whereas for female speakers the range was set at 100–500 Hz. In addition, we also assessed the influence of *perceived pitch* and *perceived speed* on listeners’ threat and intent evaluations. Such measures reflect the procedures that exist for eliciting information about speakers’ voices from ear-witnesses to potential offences, which often ask for information about an offender’s voice. Eliciting information about speakers’ voices is also a part of documents specifically relating to the evaluations of spoken bomb threat utterances, such as the UK National Counter Terrorism Security Office bomb threat checklist (National Counter Terrorism Security Office, 2016). This document asks users for information about both vocal pitch and speaking tempo (see Figure 1), albeit in a way which is both linguistically uninformed and somewhat unclear (Watt & Brown, 2020).

Participants provided their ratings in a quiet environment and used Sennheiser HD215 closed-cup headphones to listen to the voice recordings. Throughout the experiment, participants were presented with voice stimuli binaurally and were asked to answer a series of questions about each voice they heard. The volume controls on the lab PCs were standardised at 50% and no participants reported any issues with respect to hearing the audio. In order to provide a forensically relevant context to the experiment, participants were instructed that the recordings they would hear were from calls made to emergency service operators. For each recording, participants were asked to assess how high-pitched and how fast each speaker’s voice sounded on a scale from 0 (*very low-pitched/very slow*) to 100 (*very high-pitched/very fast*). They were then asked to make two separate judgements, one of threat and another one of intent to harm conveyed by the speaker using a scale from 0 (not at all threatening/no intent to harm) to 100 (extremely threatening/certain intent to harm). A rating of 0 represented *not-at-all threatening* or *no intent to harm*, and a rating of 100 represented *extremely threatening* or *certain intent to harm.* Participants were free to listen to each recording as many times as they wished to; however, they were instructed to rely on their initial “gut feeling,” rather than spending too much time thinking about their ratings. Evaluations took place immediately after exposure to each stimulus as we did not want memory to be a factor in the voice evaluation process.

### Results and discussion

#### Effects of voice characteristics on threat and intent to harm judgements

Statistical analysis probing the effects of the chosen variables on listener judgements of threat and intent to harm was conducted using random-intercept linear mixed effects regression models (hereafter lmer) constructed using the *lme4* package (Bates et al., 2015) in *R* (R Core Team, 2015). Main effect *p*-values were calculated via likelihood ratio model comparisons tests, using the *anova* function in R. This process followed the procedure outlined by Winter (2014) and involved the statistical comparison of two models—one that includes the variable under investigation, in addition to random effects, and a reduced model that excludes that same variable.^1^ This method was used separately for threat and intent to harm judgements. In each model, listener ratings (of either threat or intent to harm) formed the dependent variable, with *median F0, F0 range, speaker accent, utterance, emphasis pattern, perceived pitch, perceived speed*, and *speaker sex* included as fixed effect predictor variables. Given that the experiment involved multiple speakers and multiple listeners, *listener* and *speaker* were also included as random effects. Table 1 displays the output of both lmer models.

**Table 1.** Effects of voice characteristics on listener evaluations of threat and intent to harm in Study 1.

	Threat			Intent to harm		
	χ^2^	*df*	*p*	χ^2^	*df*	*p*
Fixed effects						
Median F0	0.40	1	.53	0.001	1	.99
F0 range	2.23	1	.14	2.66	1	.10
Speaker accent	3.58	2	.17	1.59	2	.45
Utterance	0.53	1	.47	0.03	1	.87
Emphasis pattern	3.36	1	.07	3.36	1	.11
Perceived pitch	**22.98**	**1**	**<.001**	**13.48**	**1**	**<.001**
Perceived speed	2.86	1	.09	3.21	1	.07
Speaker sex	0.22	1	.64	1.67	1	.20
Random effects						
Listener	**214.82**	**1**	**<.001**	**350.61**	**1**	**<.001**
Speaker	**7.71**	**1**	**.005**	**8.76**	**1**	**.003**

Significant effects are displayed in bold.

**Figure 2.**
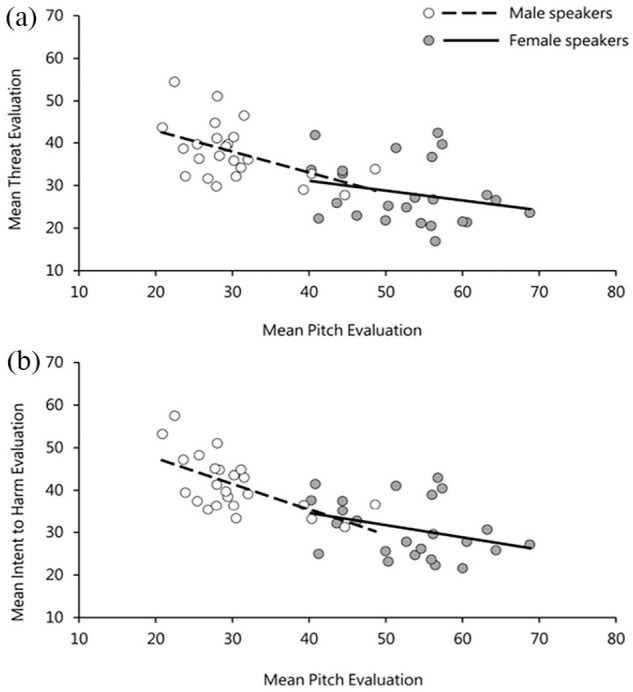
(a) Relationship between perceived pitch and threat evaluations and (b) between perceived pitch and intent evaluations in Experiment 1. Points are averaged across listener for each utterance and split in accordance with speaker sex.

The results in Table 1 highlight a significant effect of *perceived pitch* on listeners’ perceptions of both threat and intent to harm. No other variable had a significant effect on listeners’ judgements of either threat or intent to harm. The relationship between listeners’ judgements of perceived pitch and both threat and intent to harm ratings is plotted in Figure 2. Due to the pre-existing sex differences in vocal pitch, this was done separately for male and female speakers. The plots reveal that male voices with lower perceived pitch were judged as sounding significantly more threatening as well as more intentful to cause harm (*r* = −.49, *p* = .01) and (*r* = −.61, *p* = .001), respectively. This pattern, however, was not observed for female voices, (*r* = −.24, *p* = .25) for judgements of threat and (*r* = −.32, *p* = .11) for judgements of intent to harm.^2^

In addition to testing for the significance of the fixed effect predictors, we also analysed the random effects of *speaker* and *listener* within the model. Table 1 shows significant effects for both *listener* and *speaker* on evaluations of threat and intent to harm. This suggests that characteristics of both the “threatener” and the hearer can significantly influence how utterances are perceived with respect to the levels of perceived threat and intent to harm. While a large amount of variation being attributable to individual participants is commonplace in psycholinguistic experiments (Tagliamonte & Baayen, 2012), we consider the effect of *listener* particularly noteworthy as a guard against the notion that spoken threats are likely to be interpreted in the same way by different listeners (cf. the assertion made by Gingiss, 1986).

We also find striking similarities between the results of the threat and intent analyses, suggesting high levels of conceptual similarity. There was a strong positive correlation between ratings of threat and intent (*r* = .89, *p* < .001), which could indicate that these traits might not be so easily (conceptually) separable as the existing literature suggests.

#### Rater agreement

**Figure 3.**
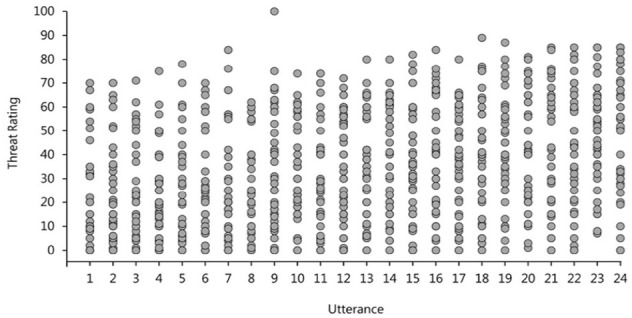
Threat ratings attributed to each of the 24 verbal threat utterances in Experiment 1. Each column represents a single utterance and each point represents a rating by a single hearer. Utterances are ranked on the *x*-axis by the mean utterance rating.

The strong effect of *listener* was rather surprising, therefore, we decided to explore this further. As there was no significant effect of stress pattern on either threat or intent ratings, we averaged across the two types of stress utterances for each speaker which gave us a total of 24 different utterances (2 per speaker—a warning and a statement). Figure 3 shows the threat ratings attributed to each of those utterances where each column represents a single utterance and each point represents a rating from a different listener. Consistent with our statistical analysis, we see a substantial amount of variance in the perceived levels of threat for each separate utterance and very little evidence for a consistent effect of utterance or speaker. It is clear that there is more variability within, rather than between the utterances. There is no clear pattern even when the utterances are ordered by mean rating, as is the case in Figure 3. Perceived threat and intent were rated on a scale from 0 to 100, which means that the reported variability in ratings attributed to the same utterance could, at least in part, be caused by perceivers using the rating scales in different ways. In order to address this, we normalised all threat and intent ratings, separately for each participant by subtracting each perceiver’s average threat/intent score from each of their individual ratings and dividing that difference by the standard deviation of their ratings. This procedure allowed us to eliminate the variability associated with differences in the use of the rating scales. Figure S1 in the Supplementary Materials plots these normalised ratings the same way as in Figure 3 and demonstrates the same pattern of results which implies that our findings reflect a true disagreement in threat attribution rather than an artefact of rating scale use.

Such findings imply that listeners perceive verbal threats in a different way and may not necessarily agree with one another. This runs counter to the large literature on rater agreement in social evaluation of both face and voice stimuli, showing that judgements of many characteristics are quite consistent (Todorov et al., 2009; Willis & Todorov, 2013; Zuckerman & Driver, 1989).

In order to further assess the between-listener variability in the data and its potential application to real-world situations, we used a permutation test, separately on each utterance in the dataset, using 1,000 random samples of 12 listeners. This was done in order to analyse the amount of variation within any given subset of listeners, and random samples of 12 listeners were chosen as this is the number of people required to sit on a jury panel in the United Kingdom. Given that juries are instructed to reach a unanimous decision in criminal cases in UK courts, it was considered interesting to see how varied listeners’ threat evaluations would be within any random set of 12. These tests were conducted using MATLAB, with 1,000 random permutations of 12 listeners for each utterance.

The analysis showed a high level of variation between listeners’ evaluations of how threatening speakers sounded. The data show that the average interquartile range for all 24 utterances extended beyond 27% of the 100-point rating scale. The lowest average threat score range across the 1,000 random trials of 12 listeners was 58, which equates to 58% of the total scale available to listeners, while the highest average threat score range was 77, which equates to more than three quarters of the total available scale. These values illustrate the high overall level of disagreement among listeners within the random samples of 12 created for this analysis. Ratings of intent presented a very similar pattern of results both in terms of rating variability (see Figure S2 for mean intent scores and Figure S3 for normalised intent scores in the Supplementary Materials) and agreement across random sets of 12 listeners.

Overall, the results of Study 1 show that the perception of both threat and intent to harm in voices is driven by the perceived rather than the measured F0. This is an important distinction given that most studies aiming to investigate the effects of vocal pitch generally use measured F0 values rather than collect perceived pitch ratings. We also show a significant lack of agreement in threat and intent ratings, emphasising that agreement cannot be assumed when people are required to provide retrospective evaluations of threats and are not either the original speaker or recipient of the threat.

## Study 2: perception of threat and intent to harm in voices and faces

Having explored the relationship between various aspects of speakers’ voices and voice-based perceptions of threat and intent to harm in Study 1, we next examined participants’ inferences when they were exposed to static face images alongside speakers’ voices. This study aimed to complement the work in Study 1 and to explore the influence of audio-visual integration in forensically relevant social evaluations. Given the known links between dominance and traits such as threat and aggressiveness (Ohala, 1984; Oosterhof & Todorov, 2008), alongside the core role that dominance perception has been reported to play in social evaluations of both faces and voices (McAleer et al., 2014; Oosterhof & Todorov, 2008), we specifically assessed whether perceived facial dominance would influence participants’ judgements of threat and intent to harm. Given the evidence for the automatic integration of facial and vocal cues with respect to dominance evaluation (Mileva et al., 2018), we hypothesised that differences in perceived dominance from people’s faces could, in turn, influence evaluations of threat and intent to harm when listeners were presented with both modalities simultaneously. As cross-modal integration occurs even when participants are instructed to ignore one of the channels (Mileva et al., 2018), we expect to observe this integration here, in the absence of any instructions to base decisions on the perceived characteristics of the facial images. Moreover, this also provides us with an opportunity to test the idea that judgements of dominance reflect our evaluation of someone’s ability to harm us rather than their intent to do so (Oosterhof & Todorov, 2008). If this is the case, facial dominance would have a significant effect on threat but not on intent to harm ratings.

### Method

#### Participants

Face–voice pairings were rated by 49 native British English-speaking participants (6 male, median age = 18, age range = 18–50). They were all students and staff from the University of York. Participants who had already taken part in Study 1 were not recruited for the present study in order to ensure they had not been pre-exposed to any of the vocal stimuli. All participants received payment or course credits for their participation. Informed consent was provided prior to participation and the study received ethical approval from the ethics committees of the Departments of Psychology and Language and Linguistic Science at the University of York.

#### Materials

The audio materials used in Study 2 comprised a subset of the audio recordings used in Study 1, specifically, the ones with emphasis placed on the word “will” (*n* = 24). This resulted in two utterances per speaker being used in the study, representing the three accent groups (8 utterances per accent) and speaker sex (12 male and 12 female utterances).

Each vocal stimulus was paired with a face selected from a pre-rated set (see Mileva, Young et al., 2019 for additional details). The original set included four images of 40 unfamiliar identities (a total of 160 images). These were all foreign celebrities, who were unfamiliar to UK participants. All images were collected with Google Image Search by typing the name of the identity and downloading the first four images that were in full colour, broadly frontal, and with no part of the face obscured by clothing or other accessories. Other than these restrictions, the images were all naturally occurring and captured a good amount of face variability from lighting, camera angle or emotional expressions. We removed any background information in the images so that only the face was seen by participants.

All images were pre-rated for dominance on a nine-point scale by an independent sample of 27 participants (3 male, mean age = 22 years, age range = 18–30). We calculated the differences in mean dominance ratings between each of the same-identity images, and selected the two images for each identity which displayed the greatest difference in mean dominance ratings. From these 40 pairs of images, the 6 male pairs with the greatest mean dominance rating difference (mean difference = 1.14, range = 0.81–1.63) and the 6 female pairs with the greatest mean dominance rating difference (mean difference = 1.45, range = 1.11–1.82) were paired with the voice recordings. Each speaker was assigned a facial identity, with consistency maintained in terms of age and sex given that the facial and vocal identities were based on images and audio samples of different people.

**Figure 4.**
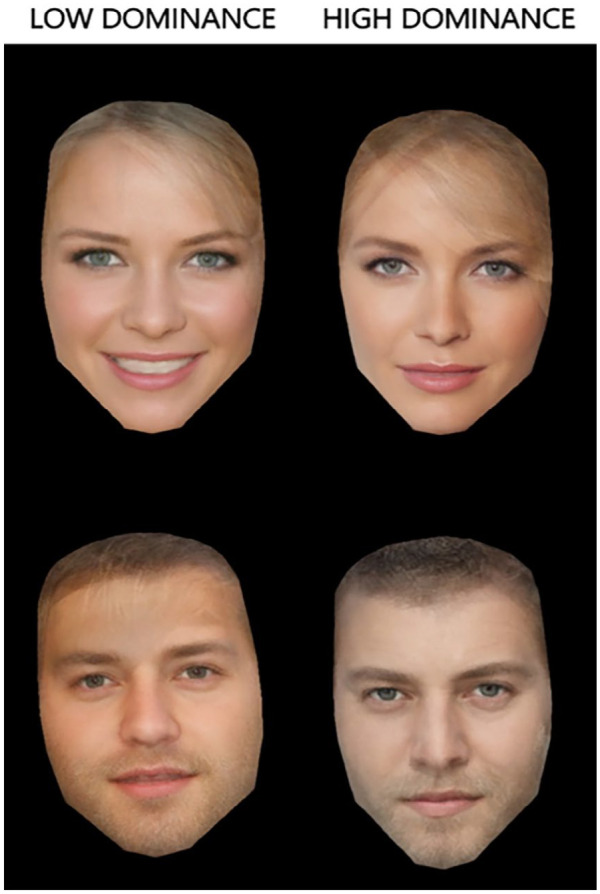
High and low dominance averages for both female and male identities, showing the facial information that guides the perception of dominance.

Rather than providing examples of the face stimuli (which is not possible due to copyright restrictions), Figure 4 shows the high and low dominance average images, separately for female and male identities. These were created by morphing all female/male images rated as high or low in perceived dominance together using the InterFace software (Kramer et al., 2018). Further details about the exact morphing procedures can be found in Mileva, Kramer et al., 2019. Crucially, this averaging technique has been used to reveal some of the facial information that drives the perception of different stereotypes or first impressions traits (Oldmeadow et al., 2013; Sutherland et al., 2013). Comparing the low and the high dominance averages makes it clear that emotional expressions play an important role in the dominance perception of female but not male faces. The overall colour warmth of the picture seems to be important for male images, with warmer tones perceived as less dominant and cooler tones perceived as more dominant. Finally, a slimmer face, the presence of make-up (for female identities), and a more direct gaze also seem to be key for the perception of dominance within our chosen image set.

#### Procedure

The experiment was created and hosted on the online platform Qualtrics (Provo, UT), however, participants completed the task in a computer lab, in person. Prior to providing their ratings, participants were instructed that they would hear calls made to emergency service operators and that they would be presented with an image of the speaker alongside hearing their voice. This follows the procedure used in Mileva et al. (2018). Other than the addition of the speaker’s face, the study followed the exact same procedure as in Study 1. Crucially for the current experiment, the questions asking listeners to infer how threatening each speaker sounded and how much intent to harm was conveyed through his or her speech referred solely to the speaker’s voice. This was done on the basis that any effect of facial dominance on listener perceptions of threat or intent to harm would be brought about by automatic audio-visual integration rather than as a result of the experimental instructions. All face–voice pairings (*n* = 48) were split into two equal subsets (*n* = 24) and each participant was randomly assigned to one of those sets. Trials were counterbalanced so that participants did not see the same image or hear the same vocal stimulus twice. The order in which the stimuli were presented was also randomised individually for each participant.

### Results

#### Effects of voice characteristics on threat and intent to harm judgements

Two random-intercept linear mixed effects models (one for threat and one for intent to harm judgements) were constructed with *median F0, F0 range, speaker accent, utterance, perceived pitch, perceived speed, speaker sex, and facial dominance* as fixed effects, and with both *perceiver* and *speaker* included as random effects. To assess facial dominance, we used the mean dominance rating assigned to each face image from Mileva, Young et al., 2019. As in Study 1, statistical significance was assessed using likelihood ratio model comparison tests. These results are displayed in Table 2.

**Table 2.** Effects of voice characteristics on listener evaluations of threat and intent to harm in Study 2.

	Threat			Intent to harm		
	χ^2^	*df*	*p*	χ^2^	*df*	*p*
Fixed effects						
Median F0	0.002	1	.96	0.70	1	.40
F0 range	2.62	1	.11	1.68	1	.19
Speaker accent	1.66	2	.43	5.49	2	.06
Utterance	**8.70**	**1**	**.003**	**6.40**	**1**	**.01**
Perceived pitch	**18.50**	**1**	**<.001**	**15.08**	**1**	**<.001**
Perceived speed	**11.19**	**1**	**<.001**	**6.72**	**1**	**.009**
Speaker sex	2.57	1	.11	0.36	1	.55
Face dominance	**14.35**	**1**	**<.001**	**4.24**	**1**	**.03**
Random effects						
Listener	**225.21**	**1**	**<.001**	**427.51**	**1**	**<.001**
Speaker	**25.643**	**1**	**<.001**	**11.92**	**1**	**<.001**

Significant effects are displayed in bold.

**Figure 5.**
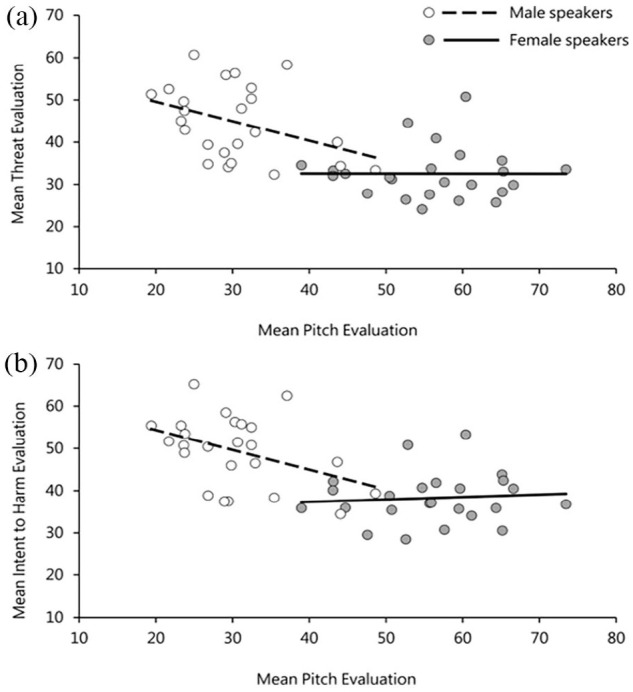
(a) Relationship between perceived pitch and threat evaluations and (b) between perceived pitch and intent evaluations in Experiment 2. Points are averaged across listener for each utterance and split in accordance with speaker sex.

Consistent with the results from Study 1, there was a significant effect of *perceived pitch* on evaluations of both threat and intent to harm. These effects are plotted in Figure 5, and, as before, show the trend for speakers perceived to have lower-pitched voices to be evaluated as sounding more threatening and conveying more intent to harm compared with speakers with higher-pitched voices. Moreover, there were clear gender differences where this relationship was only seen for male (threat ratings: *r* = −.38, *p* = .07; intent ratings: *r* = −.41, *p* = .04), but not female speakers (threat ratings: *r* = .002, *p* = .99; intent ratings: *r* = .08, *p* = .70).

**Figure 6.**
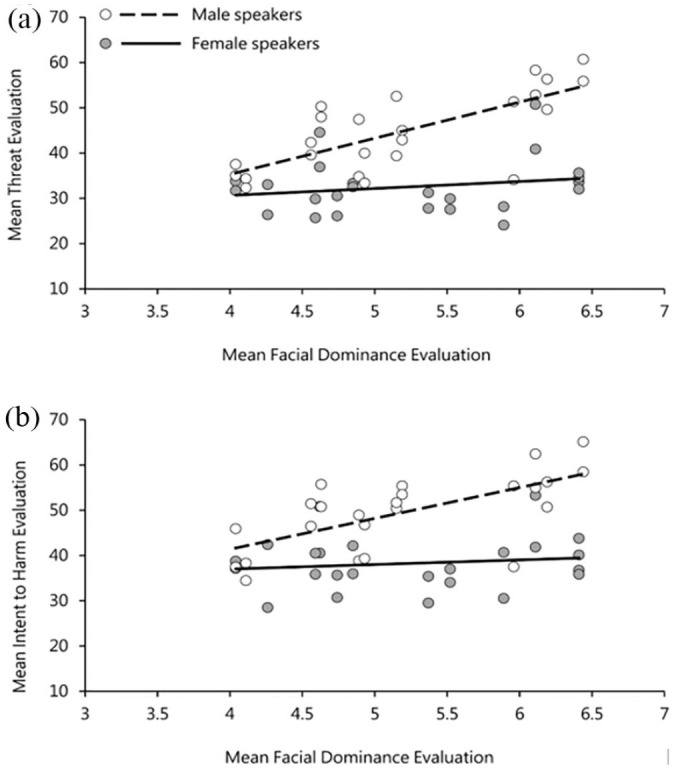
(a) Relationship between facial dominance ratings and threat evaluations and (b) between facial dominance ratings and intent evaluations in Experiment 2. Points are averaged across listener for each utterance and split in accordance with speaker sex.

Table 2 also shows a significant effect of facial dominance (dominant faces vs. non-dominant faces) on perceivers’ evaluations of threat and intent to harm. This effect was in line with our expectations that voices paired with dominant faces would be assigned higher threat ratings than those paired with non-dominant faces. Figure 6 plots the relationship between face dominance ratings and both threat and intent to harm judgements separately for male and female speakers. This effect was, again, seen for male (threat: *r* = .72, *p* < .001; intent: *r* = .65, *p* < .001), but not female (threat: *r* = .20, *p* = .34; intent: *r* = .13, *p* = .51) identities.^3^ Critically, it also existed in the absence of any explicit instruction to use facial stimuli as a basis for evaluations.

Finally, both the perceived speech rate and the type of utterance had a significant effect on listeners’ perceptions of threat and intent to harm. Our results demonstrated that warning utterances and voices which were perceived to be slower were perceived to sound more threatening and intentful than statement utterances and voices that were perceived as more fast-paced. These variables did not influence perceivers’ evaluations significantly in Study 1. This might be due to the removal of emphasis pattern as a predictor of threat and intent which could have allowed for the effect of utterance and/or perceived speed to explain a larger proportion of the variance. The difference in the importance of this factor might also reflect the presence of a face image which could have increased the attention to the target and what they were saying (i.e., their utterance).

As was the case in Study 1, we also used likelihood ratio model comparison analysis to test for the significance of the random effects of *perceiver* and *speaker* on evaluations of threat and intent to harm. Replicating our previous findings, we show that both perceiver and speaker had a significant influence on judgements of threat and intent to harm. These effects reflect that a significant proportion of the variance in the data was not captured by the fixed effects, and instead was attributable to random variations between individual speakers and individual perceivers. As in Study 1, here we also find a very similar pattern of results for ratings of threat and intent to harm, suggesting that they are conceptually related to a substantial extent. In fact, the correlation between ratings of threat and intent (*r* = .97, *p* < .001) in Study 2 was significantly stronger compared with that found in Study 1 (*Z* = 2.54, *p* = .006), implying that the presence of a face might be strengthening their conceptual association.

#### Rater agreement

**Figure 7.**
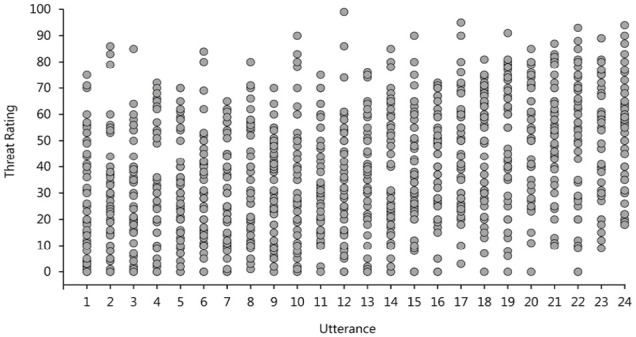
Threat ratings attributed to each of the 24 verbal threat utterances in Experiment 1. Each column represents a single utterance and each point represents a rating by a single hearer. Utterances are ranked on the x-axis by the mean utterance rating.

Finally, we explored the variability in threat and intent ratings as well as the perceivers’ judgement consensus. Figure 7 shows threat ratings attributed to each utterance where each column represents a single utterance and each point represents a rating from a different perceiver. Consistent with the findings from Study 1, we see a comparable amount of variance in the judgements attributed to each utterance. The fact that that there is no clear pattern of results even when utterances are ranked by their mean rating, as is the case in Figure 7, supports our argument that there was actually very little agreement in the perception of threat and intent to harm among perceivers in this experiment.

We also ran the same permutation tests using 1,000 random samples of 12 perceivers as in Study 1, in an attempt to demonstrate the effect of this variability on perceiver subsets that were the same size as a UK jury panel. The analysis showed comparable values to those in Study 1. The average interquartile range, for all 24 utterances, extended beyond 27% of the 100-point rating scale. The lowest average threat score range across the 1,000 random iterations was 55, which equates to 55% of the total scale available to perceivers, while the highest average threat score range was 76, which equates to more than three-quarters of the total available scale. Ratings of intent presented a very similar pattern of results both in terms of ratings variability (see Figure S6 in Supplementary materials) and agreement across random sets of 12 perceivers. Overall, such results provide little evidence for any agreement in the perception of threat and intent to harm and highlight the need to investigate these forensically important traits further.

## General discussion

The present studies aimed to address our current lack of understanding concerning how people perceive a so-called “threatening tone of voice.” We explored how a number of measures, including F0, intonation, speaker accent, emphasis pattern, perceived pitch, perceived speech speed, and speaker sex, as well as the visual information provided by the speaker’s face, can influence decisions about the perceived threat and intent in a given utterance. Our findings show a consistent effect of perceived pitch, where low-pitched voices were perceived as more threatening than high-pitched voices (specifically for male speakers) and a strong effect of perceived facial dominance. Moreover, we demonstrate that listeners do not necessarily agree on their threat and intent evaluations, which is an important finding considering that people can be asked to evaluate threats as part of evidence in courtrooms.

The most consistent effect across both experiments was that of *perceived pitch* on listeners’ judgements of threat and intent to harm. The finding that voices perceived to be lower in pitch were generally evaluated as sounding more threatening than those perceived to be higher pitched supports previous research identifying a link between lowered pitch and the perception of dominance, aggression and other related traits (Mileva et al., 2018; Ohala, 1984; Puts et al., 2006, 2007; Tusing & Dillard, 2000). In both studies, however, the relationship between perceived pitch and threat or intent judgements was observed for male, but not female, speakers.

Much of the previous literature on the role of vocal pitch in social evaluation has been traditionally based on male voices only, with only a few that also consider female voices. These latter studies present very inconsistent findings with some showing that lower pitch leads to higher dominance ratings for both male and female speakers (Borkowska & Pawlowski, 2011; Jones et al., 2010; Mileva et al., 2018), whereas others show this effect for male voices only (Tsantani et al., 2016) or even the reverse pattern for female voices, with high-pitched voices perceived as more dominant (McAleer et al., 2014). There are many methodological differences among these studies that could be driving the inconsistent findings such as the nature of the task (e.g., a 2AFC task or a rating task), the type of stimuli used (e.g., meaningful utterances, reversed speech or vowel sounds), or adopting a particular context (e.g., selecting a political leader or a romantic partner). In fact, there is already some evidence that social context can influence our preference for low or high pitch (Vukovic et al., 2008).

Regardless of the potential reasons, it seems that the link between low pitch and the perception of high dominance is much more stable and pronounced for male rather than female speakers and this aligns with our findings given the close links between the perception of threat and dominance (Oosterhof & Todorov, 2008). One explanation for these findings involves the way dominance might be interpreted for male and female voices. It is possible that perceivers are rating physical dominance when presented with male voices, but social dominance when presented with female voices. This is consistent with Puts et al. (2007), who find that vocal pitch is much more related to the perception of physical, rather than social dominance. Moreover, a much simpler explanation that we need to acknowledge concerns the higher proportion of female perceivers in our sample. Thus, it is possible that pitch might have a stronger effect on the perceived dominance of female faces with a more gender-balanced sample. However, while there is some evidence for rater gender differences in first impressions from faces (Mattarozzi et al., 2015), no such systematic differences have been shown for judgements attributed to voices.

In our studies, perceived pitch was a much stronger predictor of threat judgements than the average F0, which illustrates the potential importance of engaging with listeners’ subjective perceptual scales. This is consistent with Tompkinson and Watt (2018), who showed only a small-to-medium sized correlation between median F0 and listeners’ own pitch evaluations. This was true even after standardising the pitch scores and considering the effect of other acoustic measures which could have influenced judgements. Such findings have important implications for both theory and practice. Contemporary voice perception models as well as the great many studies on varied aspects of vocal pitch currently fail to consider the effects of listeners’ subjective evaluations of pitch. This may leave important influences of vocal pitch undetected and our understanding of voice perception incomplete. Moreover, listeners’ subjective experiences are sometimes all that is available in an applied context of ear-witness evidence. This is another example of the discrepancy between theoretic research and applied forensic needs.

In addition to examining the influence of aspects of a speaker’s voice, we also addressed the question of whether perceived facial dominance would influence listeners’ judgements of threat and intent. This was in spite of the fact that participants were asked to judge how threatening speakers *sounded*, not how threatening they *looked* or how threatening they *were*. The results of Study 2 showed that perceived facial dominance did influence listener evaluations of how threatening speakers sounded, even in the absence of any explicit instruction to listeners that they should base their evaluations on both facial and vocal information. This finding provides support for the view that the integration of audio-visual information in social evaluations of people is, to some degree, involuntary (Mileva et al., 2018) and can occur even without the temporal integration of the two types of cues (i.e., a static face paired with a dynamic voice recording).

In Study 2, voice recordings were paired with static, rather than dynamic, faces which might not be an optimal representation of reality. While there are some studies demonstrating the importance of dynamic face and voice presentation, they are mostly focused on different processes such as speech recognition (Munhall et al., 1996) or limited to the perception of familiar identities (Robertson & Schweinberger, 2010; Schweinberger, 2013). In fact, studies which compare static and dynamic faces report no significant effects of animation or temporal synchrony (Schweinberger et al., 2007). Moreover, there is evidence for audio-visual integration with static faces in studies of emotion recognition (De Gelder & Vroomen, 2000) and, more importantly, social evaluation (Mileva et al., 2018) where participants’ responses were significantly influenced by the presentation of a static face even after instructions to focus on the voice instead. We observed a similar pattern here as the perceived dominance of the face stimulus influenced the threat ratings of the voice recordings despite the voice-focused phrasing of the judgement questions (e.g., How threatening does this speaker sound?). Such findings imply that pairing a static face with a voice recording might be sufficient to produce audio-visual integration, although a dynamic, synchronised display might further facilitate this integration and amplify the effect of face dominance.

Our analysis showed that facial dominance was a significant predictor of both threat and intent to harm ratings. However, while the correlation between perceived facial dominance and threat was numerically stronger than the correlation between perceived dominance and intent, this difference was not statistically significant (*Z* = 0.43, *p* = .334). This could be of theoretical importance to the underlying structure of first impressions and the current view that the fundamental first impression dimensions, trustworthiness and dominance, reflect evaluations of someone’s intent and ability to cause us harm, respectively (Oosterhof & Todorov, 2008). Following this argument, we hypothesised that facial dominance would be more closely related to the perception of threat, rather than intent. This, however, was not observed in our data, implying that the distinction between trustworthiness and dominance in terms of threat evaluation might not be as clear-cut as previously thought. Ratings of intent are usually not included in existing social evaluation models, so it is still not clear how this legally relevant trait might map onto the fundamental evaluation dimensions. Together with our findings, this further highlights the need to more systematically investigate the accepted evolutionary account of the basis of first impressions. What is more, both threat and intent present a very similar pattern of results and we show an exceptionally high correlation between them, especially in Study 2 where facial information was also available to our perceivers. Therefore, while these two concepts are clearly distinct in a more legal context, they seem to capture the same first impression judgement. This also shows the potential detachment between legal language and lay-person language, as the high levels of correlation between ratings for the two traits would suggest that participants in the experiment treated the two concepts very similarly despite them having subtle but different meanings in a more legal context (Public Order Act, 1986, Section 4A).

The theoretical accounts of first impression formation involve both social categorisation and overgeneralisation processes (Secord, 1958; Zebrowitz & Montepare, 2008). As such, they are mainly the result of interpreting traits stereotypically assigned to different social groups or characteristics of temporary states (such as emotional expressions) as signals of stable personality traits. There is already a multitude of studies demonstrating the key role of age, gender, emotional expressions, and other low-level picture properties in first impressions (see Todorov et al., 2015 and Zebrowitz & Montepare, 2008 for reviews). In fact, the influence of emotional expressions has even been observed with seemingly neutral faces, where classifications from an algorithm trained to detect subtle resemblances to emotional expressions in neutral faces were able to explain some of the variability in first impression judgements (Said et al., 2009). We can therefore expect the face images we have used in Study 2 to vary along all of these dimensions in order to produce high or low levels of perceived dominance. For example, male identities will likely receive higher dominance ratings, whereas faces displaying positive emotions will likely receive lower dominance ratings. The averages created from our face stimuli presented in Figure 2 demonstrate that emotion might be an important cue for female faces, but not so much for male faces, where lower-level properties such as colour temperature seem to guide the perception of dominance. Critically, while all of these factors could contribute to the perception of dominance, none of them vary systematically within our set, other than the dominance ratings attributed to those faces. That is, not all high dominance faces had a negative emotional expression (for example). Interestingly, in some cases a smile combined with other cues such as a more direct gaze amplified the perception of dominance, perhaps due to being interpreted in a more sinister way. This observation fits nicely with more recent work on first impressions arguing that there is value in considering the idiosyncratic ways in which people vary and how that might affect subsequent social judgements (Mileva, Young et al., 2019).

We consider the significant effects of both *listener* and *speaker* in both experiments to be an important and somewhat surprising finding which highlights the dangers that accompany assumptions surrounding the interpretation of potential language crimes such as threats. These results suggest that there is scope for disagreement among listeners about the level of threat or intent to harm in a given utterance, alongside differences among speakers that were not captured by the fixed-effect variables. In highlighting *listener*, we argue that caution should be advised around any assumption that all people will evaluate either how threatening or intentful an utterance sounds in a comparable or similar way to one another. By showing that a high level of disagreement existed between listeners evaluating the same voices, both with respect to the whole set of participants and to the random samples of 12 listeners, we argue that this work should serve to promote caution in any kind of automatic assumptions surrounding the ways in which a potentially threatening utterance will be perceived by listeners. Given that threats are inherently bound to context, this finding is more applicable to the context of third-party evaluations of threats of the kind made by jurors in courtrooms. Given that participants in this experiment were not the direct recipients of a threat, the findings should not be automatically assumed to apply to threats delivered in this context. However, we argue that the finding of high levels of disagreement between listeners in this experiment should, nevertheless, weaken any assumptions that people will automatically agree when evaluating or perceiving language crime utterances. This ties into the idea that person perception is inherently holistic and relies on not only the person being perceived, but the knowledge, attitudes, values, and experiences of the perceiver. These findings are also in stark contrast to the consistent finding of high rater agreement in the social evaluation literature (Todorov et al., 2015; Zuckerman & Driver, 1989). This could be either due to the many different ways threats can be interpreted and perceived by listeners or due to the potential overestimation of agreement following Cronbach’s alpha. Both of these possible explanations have important theoretical implications.

The lack of an effect for speaker accent was surprising given the large quantity of previous research emphasising the importance of accent in social evaluations of speakers (Coupland & Bishop, 2007; Giles, 1970; Labov, 1972; Preston, 2002; Watson & Clark, 2015). This may be attributable to the relative strength of the other tested effects, the choice of stimuli, or the use of a non-matched-guise design, which may have limited the perceptual strength of some of the accent features exhibited by speakers. It may also be that the group of listeners tested were not susceptible to bias based on the hypothesised stereotypes about the accents presented in the study.

The present set of experiments aimed to produce results relevant to both theoretical and more applied research, but the studies are not without some limitations. In order to control for contextual information presented to listeners, we based the experiments upon a real-world scenario in which there is the potential for the evaluation of how threatening unfamiliar speakers sounded, namely the evaluation of emergency service calls involving indirect bomb threats. This, we argue, struck a balance which was general enough to draw meaningful conclusions about the perception of threat and intent to harm from vocal and facial stimuli, whist also retaining some contextual control over the experiments. We should, however, note the potential for the repetitive nature of the experiment to cause some listeners to believe that the stimuli were simulated. Another caveat that should be considered is the potential carryover from ratings of pitch and speech speed to ratings of threat and intent to harm. While this might be unlikely, as we see a different pattern of results in Experiments 1 and 2, a blocked design might be able to address this issue in future studies. We also acknowledge that our sample of perceivers contained very few males, which, given the evidence for some rater gender differences (Mattarozzi et al., 2015), might limit the generalisability of our findings. However, no such systematic differences have been shown for judgements attributed to voices, and while this is consistent with many other studies in psychology and not considered to be a prohibitive problem for this research, we acknowledge the limitation and any potential effects of listener sex on perceptual judgements could be investigated in a future study.

Given the multitude of environments and contexts in which threats can be made, and the unknown and probably very large number of variables which could influence how they are perceived, it would clearly be unwise to over-generalise the findings of this study to any genuine situation involving spoken threats. However, our approach in this article was to present two studies which focused on integrated person perception in the context of a frequently occurring yet complex type of language crime. Our findings, Both in terms of the role of perceived pitch, rather than measured F0 and the lack of agreement in ratings of threat and intent to harm, should both challenge existing voice perception models and provide further understanding about the way verbal threats are interpreted and perceived. Our studies highlight the issues with examining perceptions of faces and voices holistically, while also highlighting the benefits of combining both face and voice perception to advance and improve knowledge about the information we use to form judgements of others in potentially consequential situations.

## Supplemental Material

sj-pdf-1-qjp-10.1177_17470218231169952 – Supplemental material for Perception of threat and intent to harm from vocal and facial cues Click here for additional data file. Supplemental material, sj-pdf-1-qjp-10.1177_17470218231169952 for Perception of threat and intent to harm from vocal and facial cues by James Tompkinson, Mila Mileva, Dominic Watt and A Mike Burton in Quarterly Journal of Experimental Psychology

## Notes

1 For example, to test the effect of perceived pitch on listener ratings, a model containing perceived pitch as a fixed effect is compared with the same model with perceived pitch removed as a fixed effect.

2 As part of this analysis, the potential effects of outliers were considered. We used modified *Z*-scores in order to detect any outliers in these data, with values greater than 3.5 or less than −3.5 being excluded. For Study 1, this resulted in two data points being excluded from the threat and perceived pitch set and three data points being removed from the intent and perceived pitch set for male voices. No data points were identified as outliers for female voices. Following these exclusions, we observe the same pattern of results with significant negative correlations for male voices and no significant correlations for female voices.

3 As for Study 1, the potential effect of outliers was considered as a part of this analysis. For Study 2, one data point was removed from the threat and pitch and threat and face dominance sets for female identities. No outliers were identified for male identities. Again, following these exclusions, we observe the same pattern of results as was observed when they were included in the analysis.
