# Supplementary material for: Perception of threat and intent to harm from vocal and facial cues
Source: Q J Exp Psychol (Hove). 2023 May 10;77(2):326–42. doi: 10.1177/17470218231169952 (PMC10798027; doi:10.1177/17470218231169952)
Supplement: sj-pdf-1-qjp-10.1177_17470218231169952 – Supplemental material for Perception of threat and intent to harm from vocal and facial cues [file sj-pdf-1-qjp-10.1177_17470218231169952.pdf]

Supplementary Materials

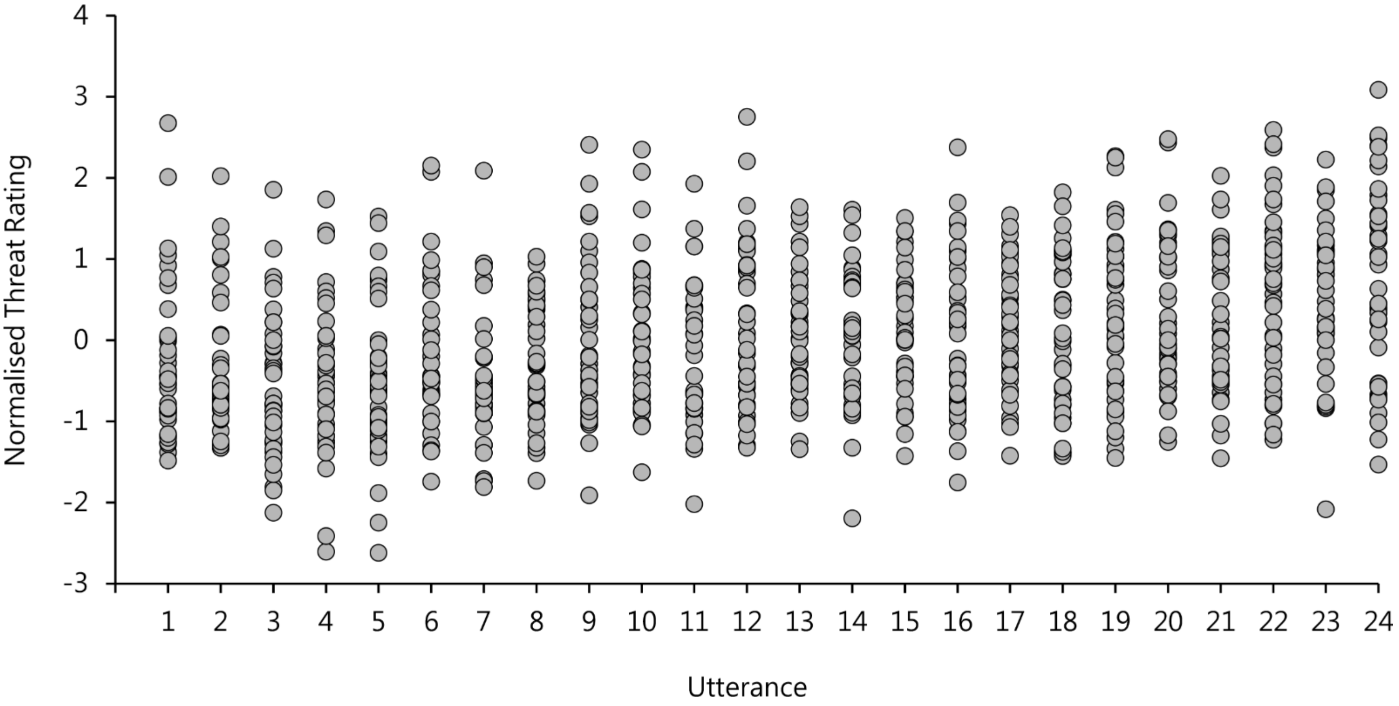

Figure S1. Normalised threat ratings attributed to each of the 24 verbal threat utterances in Experiment 1. Each column represents a single utterance and each point represents a rating by a single hearer. Utterances are ranked on the x-axis by the mean utterance rating.

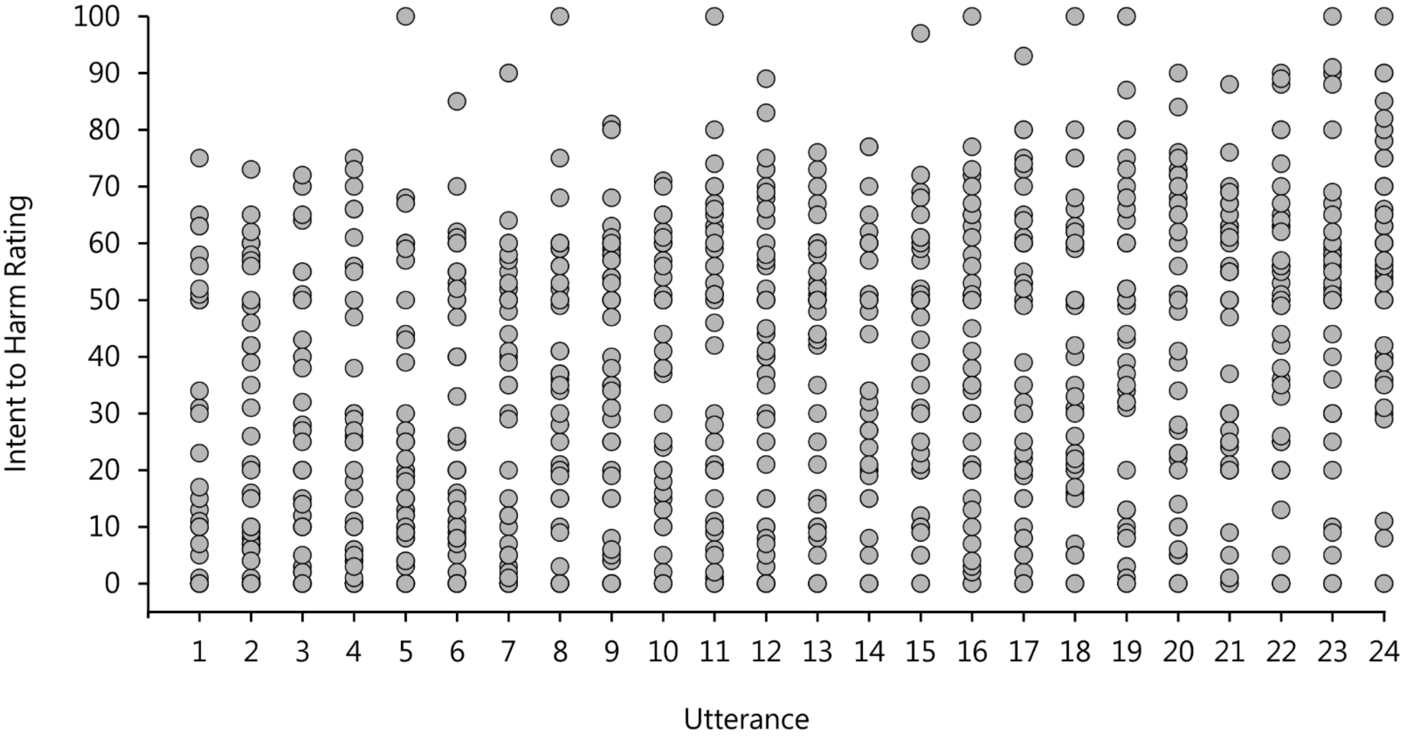

Figure S2. Intent ratings attributed to each of the 24 verbal threat utterances in Experiment 1. Each column represents a single utterance and each point represents a rating by a single hearer. Utterances are ranked on the x-axis by the mean utterance rating.

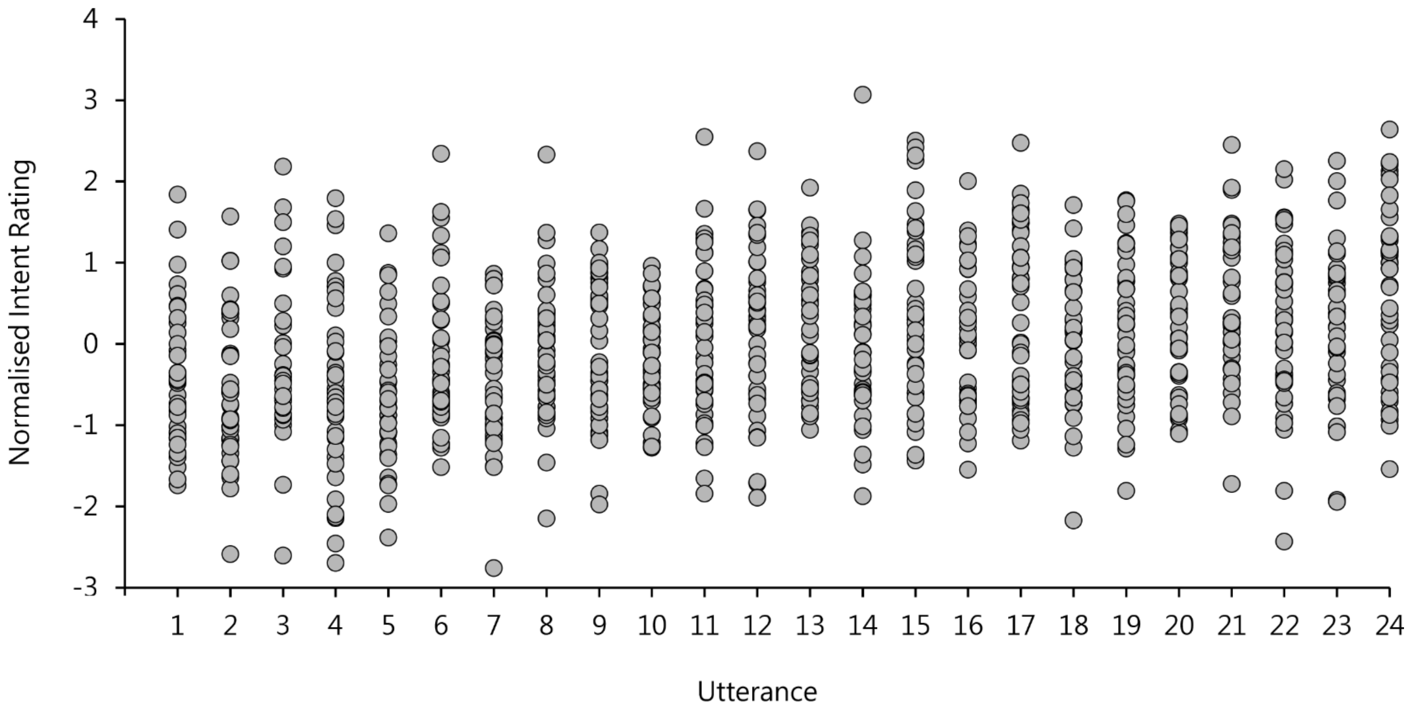

Figure S3. Normalised intent ratings attributed to each of the 24 verbal threat utterances in Experiment 1. Each column represents a single utterance and each point represents a rating by a single hearer. Utterances are ranked on the x-axis by the mean utterance rating.

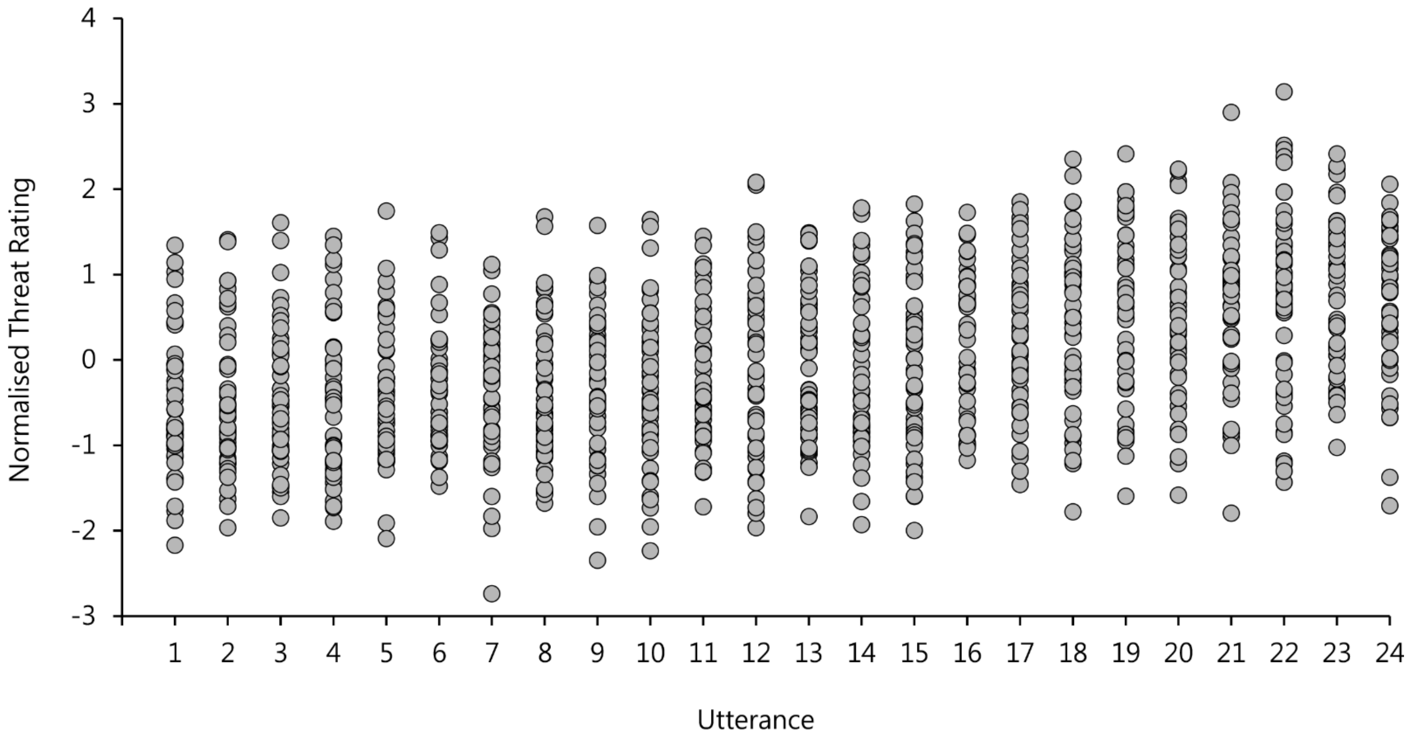

Figure S4. Normalised threat ratings attributed to each of the 24 verbal threat utterances in Experiment 2. Each column represents a single utterance and each point represents a rating by a single hearer. Utterances are ranked on the x-axis by the mean utterance rating.

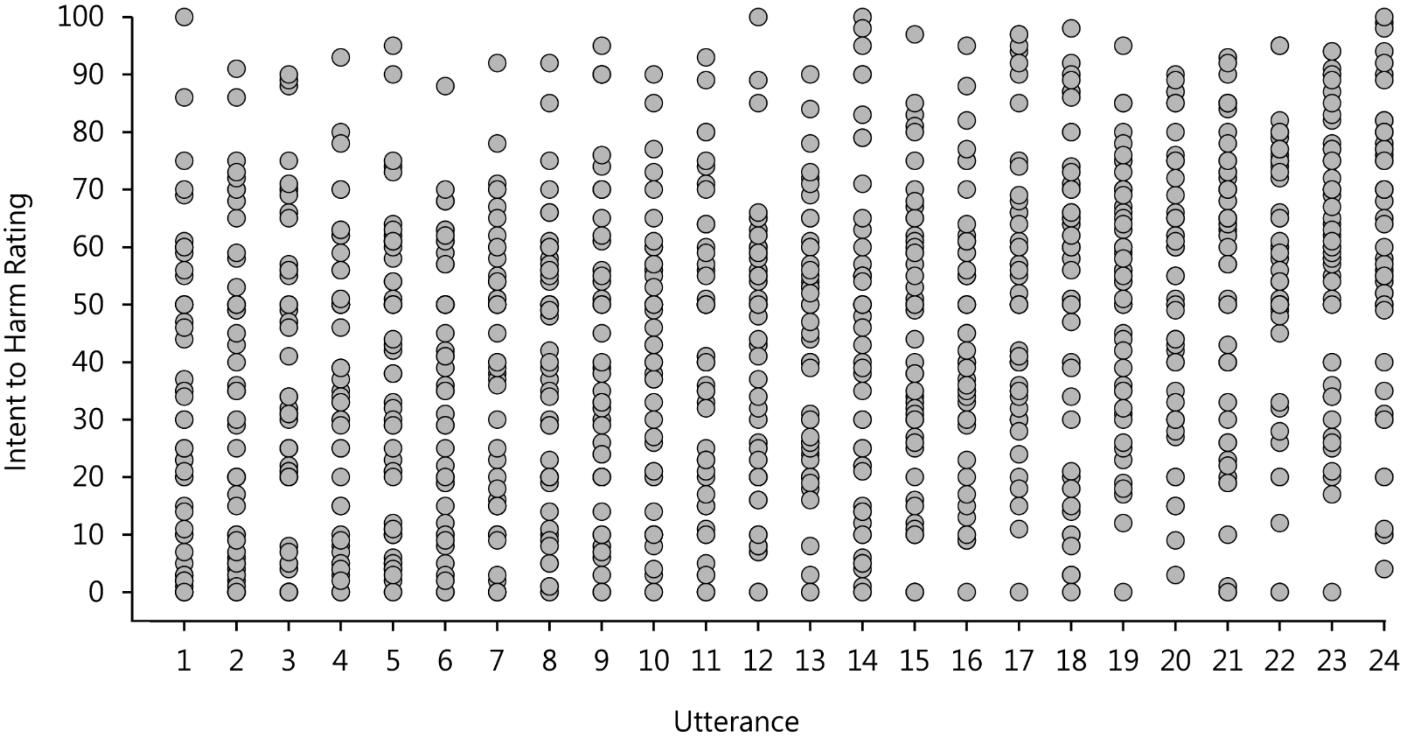

Figure S5. Intent ratings attributed to each of the 24 verbal threat utterances in Experiment 2. Each column represents a single utterance and each point represents a rating by a single hearer. Utterances are ranked on the x-axis by the mean utterance rating.

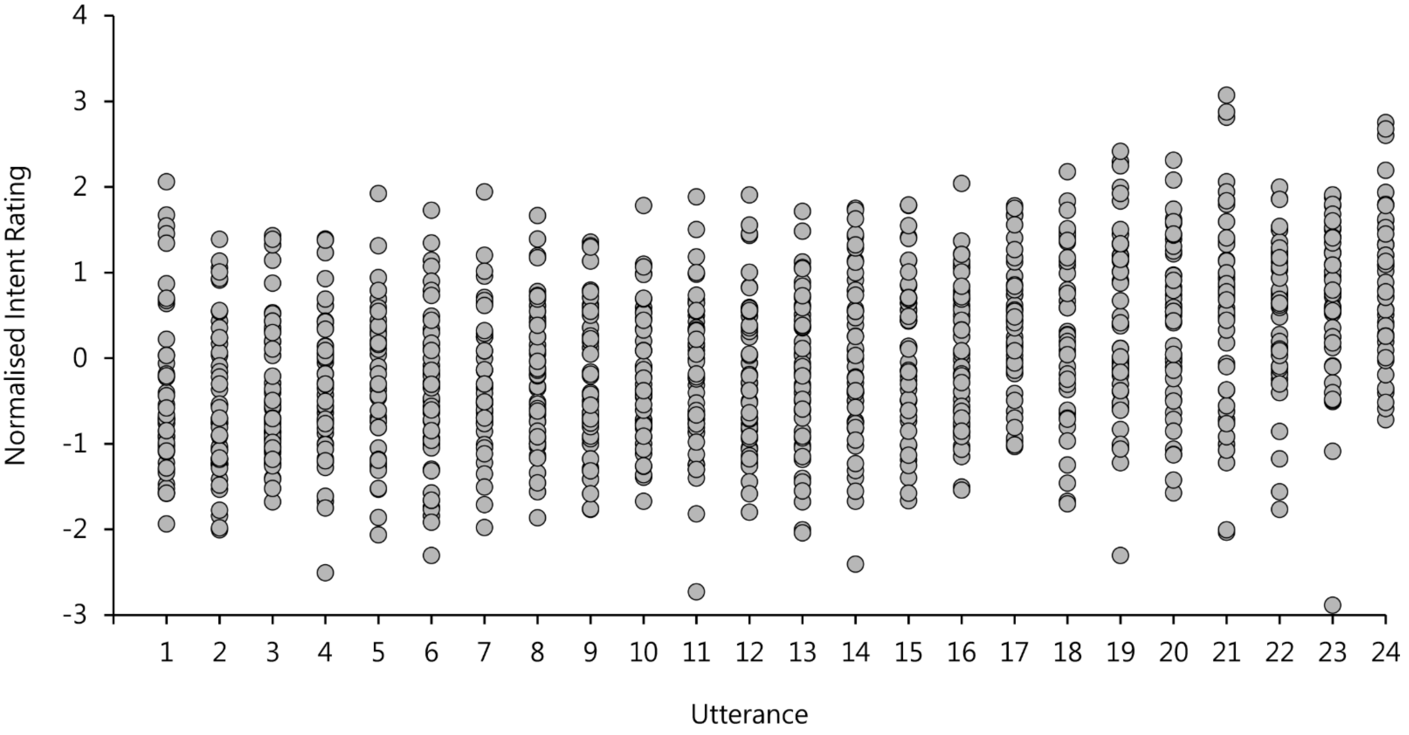

Figure S6. Normalised intent ratings attributed to each of the 24 verbal threat utterances in Experiment 2. Each column represents a single utterance and each point represents a rating by a single hearer. Utterances are ranked on the x-axis by the mean utterance rating.
